# Barriers and Predictors of Lyme Disease Vaccine Acceptance: A Cross-Sectional Study in Poland

**DOI:** 10.3390/vaccines13010055

**Published:** 2025-01-10

**Authors:** Dawid Lewandowski, Artur Sulik, Filip Raciborski, Milena Krasnodebska, Joanna Gebarowska, Aleksandra Stalewska, Kacper Toczylowski

**Affiliations:** 1 Department of Pediatric Infectious Diseases, Medical University of Bialystok, 15-274 Bialystok, Poland; 2 Department of Environmental Hazard Prevention, Allergology and Immunology, Warsaw Medical University, 02-007 Warsaw, Poland

**Keywords:** Lyme disease, vaccine acceptance, vaccine hesitancy, public health, tick-borne diseases, vaccination attitudes, Poland, preventive medicine, healthcare trust

## Abstract

Background/Objectives: Lyme disease (LD) is a major public health problem in Europe and the United States, with increasing incidence and not many prevention options. Vaccine hesitancy might be a significant barrier to successful vaccination campaigns having in mind previous vaccine development failures. This study aimed to evaluate the public’s perception of LD vaccination in Poland, assess willingness to vaccinate, and identify factors influencing vaccination attitudes. Methods: A cross-sectional survey was conducted among parents of children hospitalized at the University Children’s Hospital in Bialystok, Poland. The survey consisted of 29 questions regarding demographics, LD knowledge, vaccine attitudes, and perceived risks. Data were collected between January and December 2023 and analyzed using descriptive and inferential statistics to identify predictors of respondents’ positive vaccination attitudes. Results: A total of 503 valid responses were analyzed. Most respondents (72.4%) showed positive attitudes towards vaccination, while 18.5% were neutral and 9.1% were negative. Trust in health experts emerged as an important predictor of vaccination acceptance (OR 22.84; *p* < 0.001). More than 80% of participants recognized an LD vaccine as necessary, and 64.21% believed it would reduce their concerns about LD. Willingness to vaccinate was influenced by general positive vaccine attitudes, recognized danger of LD, and belief in the vaccine’s ability to ease fears. Notably, 40.8% of respondents were uncertain about vaccine risks, with this group tending to be younger, less educated, and expressing lower trust in medical professionals. Conclusions: Public perception of LD in Poland indicates a high acceptance of a potential LD vaccine. Still, addressing vaccine hesitancy remains critical, particularly among undecided or neutral respondents. Building trust in healthcare professionals and addressing safety worries are important to increasing future LD vaccine use.

## 1. Introduction

Lyme disease (LD) is the most common tick-borne disease in the United States and Europe [1]. The disease was first recognized in the late 20th century, and since then, the number of LD cases has been increasing [2]. The main vector of LD in Europe is *Ixodes ricinus*, an arachnid that is especially common in the northeastern part of Poland [3,4]. The tick transmits *Borrelia* spirochetes, which are responsible for the symptoms of Lyme disease. In Europe and Asia, Lyme disease is mostly caused by two *Borrelia* genospecies—*B. afzelii* and *B. garinii*, resulting in regional variations in disease manifestations. Overall, eleven species from the *Borrelia burgdorferi* sensu lato complex (*B. afzelii*, *B. bavariensis*, *B. garinii*, *B. japonica*, *B. lusitaniae*, *B. sinica*, *B. spielmanii*, *B. tanukii*, *B. turdi*, *B. valaisiana*, and *B. yangtze*) are strictly associated with Eurasia. Meanwhile, *B. burgdorferi* sensu stricto causes LD both in the USA and in Europe [3,5,6]. The heterogeneity of *Borrelia* spirochetes is one of the reasons for the diversity of clinical symptoms of the disease, which can affect the skin, joints, heart, or the peripheral or central nervous system [4].

Preventing LD poses one of the greatest challenges in public health management. At present, tick bite prophylaxis remains the best preventive measure for the disease [7]. These prevention strategies include avoiding tick exposure, using repellents, wearing appropriate clothing, conducting a thorough tick check on the entire body, and removing ticks promptly. Our previous study showed that even respondents living in areas with high tick activity have only moderate knowledge about tick bite prevention. They are often reluctant to use repellents or prefer products made from natural ingredients without N, N-diethyl-m-toluamide (DEET) [8]. These misconceptions may be related to the commonly known side effects of using DEET [9]. For this reason, the development of a Lyme disease vaccine could be an important step forward in the prevention of the disease. However, the introduction of a new vaccine will necessitate addressing vaccine hesitancy—a delay or refusal to vaccinate. This phenomenon can be influenced by many factors, such as a lack of trust in the efficacy, safety, or necessity of vaccines, as well as other barriers, like limited access to healthcare and the perception that vaccine-preventable diseases pose minimal risk. For example, in Poland, despite widespread vaccination promotion campaigns, only 22.6 million people have been fully vaccinated against COVID-19, representing about 60% of the Polish population [10].

LYMErix™ was the first Lyme vaccine approved by the US Food and Drug Administration (FDA) in the United States in 1998 [11]. The vaccine faced significant vaccine hesitancy, largely fueled by public concerns over potential side effects. Although investigations did not find evidence linking the vaccine to harmful effects, widespread media coverage and the rise of anti-vaccine movements amplified these fears, leading to its eventual withdrawal from the market in 2002 [12,13]. This episode highlights how vaccine hesitancy, even when not supported by scientific data, can have a profound impact on public health initiatives. Recently, 20 years after the Lyme vaccine withdrawal, an attempt to develop a new vaccine has been made. The new intramuscular vaccine, which targets the outer surface protein A (OspA) of *Borrelia burgdorferi*, is currently in Phase 3 human trials [14,15]. The vaccine will be available for individuals aged 5 years and older. The previous LD vaccine failure reminds us that transparent data about the vaccine and its safety profile are crucial to avoid vaccine hesitancy. The rising incidence of LD on a global scale calls for an in-depth analysis of public sentiment regarding preventive measures. Surveys conducted among people living in LD-endemic areas and forest service employees showed that they hold favorable attitudes toward a future LD vaccine [16,17].

This study aimed to evaluate the public perception of LD vaccination, assess willingness to vaccinate, and identify factors influencing vaccination attitudes, including the role of personal or familial experiences with tick bites and LD.

## 2. Methods

### 2.1. Hypothesis

The survey aimed to explore parental attitudes toward LD vaccination, focusing on factors influencing vaccine acceptance, concerns, and perceived risks. We hypothesized that individuals with previous experiences of tick bites or Lyme disease (in themselves or close relatives) would demonstrate a greater willingness to vaccinate, which might be driven by higher perceived risk. Also, we hypothesized that vaccine hesitancy would correlate with lower trust in healthcare professionals, fear of vaccine side effects, and low perceived risk of contracting the disease.

### 2.2. Study Design

This single-center cross-sectional study aimed to evaluate parental attitudes toward LD vaccination in Poland. The study was conducted through a paper survey, targeting parents of children aged 0–18 years, hospitalized in the Department of Pediatric Infectious Diseases at University Children’s Hospital in Bialystok, Poland.

### 2.3. Survey Instrument

The survey, composed of 29 questions, was designed to assess various aspects of parental attitudes towards LD vaccination, including demographics, interest in LD, knowledge about LD, personal tick bite experience, perceived risk and severity of LD, fear of contracting the disease, general vaccination attitudes, willingness to vaccinate against LD, and expectations for an LD vaccine. Additionally, trust in health experts and concerns about vaccine safety were assessed.

The questions used a variety of formats, as follows:

1. Demographic information (closed-ended questions): Questions related to age, gender, number of children, education level, place of residence, and financial situation.
2. Experience and knowledge (Likert scales and Yes/No): Participants were asked to rate their interest in LD, assess their own knowledge, and report prior experiences with tick bites or LD.
3. Risk perception and fear (Likert scales): Perceived risk of tick bites, risk and fear of contracting LD, and severity of LD were measured on five-point Likert scales, ranging from “Very Low” to “Very High” and “Not Dangerous” to “Very Dangerous”.
4. Vaccine attitudes and intentions (Likert scales): Questions addressed participants’ perceived need for an LD vaccine, willingness to vaccinate, impact of an LD vaccine on fear of the disease, fear of vaccine side effects, general attitude towards vaccines, and trust in health authorities. The perceived need for an LD vaccine was measured through three questions: (1) The perceived sufficiency of preventive measures such as repellents and body checks, (2) The belief that antibiotic treatment alone is sufficient to negate the need for vaccination, and a direct question about (3) How necessary participants consider an LD vaccine.
5. Expectations and vaccine characteristics (Likert scales): Respondents were asked to indicate the importance of various potential attributes of an LD vaccine, such as efficacy, safety, duration of protection, ease of access, and cost. They also specified the maximum amount they would be willing to pay for a complete vaccination cycle.

Vaccine intention was measured through multiple items, with a primary indicator being the direct question: “Would you be willing to vaccinate yourself if an LD vaccine was available?” and “Would you be willing to vaccinate your child if an LD vaccine was available?”, scored on a five-point Likert scale ranging from “Definitely not” to “Definitely yes”. Participants’ overall stance toward vaccination was also captured by the question: “What is your general attitude towards vaccination?”, allowing for a classification of positive, neutral, or negative vaccine propensity.

### 2.4. Validation and Reference to Existing Studies

The survey items were developed based on existing literature on vaccine hesitancy and disease risk perception. Key constructs, such as perceived severity, fear of disease, and vaccine safety concerns, align with validated scales used in previous studies on vaccine acceptance (e.g., influenza, HPV, and TBE) [8,18,19,20]. While some questions were adapted to reflect the specific context of Lyme disease, they were rooted in established psychological and public health frameworks.

The quality of this survey was evaluated by a small (*n* = 20) pilot phase, during which we distributed the first versions of the survey among the hospital staff. After collecting feedback, we improved the quality of the survey and prepared the final version of the questionnaire. Translated questions are available in the Supplementary Materials.

### 2.5. Data Collection and Sample Characteristics

The survey was administered over a period of one year (January 2023–December 2023). Invitations to participate were distributed among parents of children hospitalized in the Department of Pediatric Infectious Diseases at University Children’s Hospital in Białystok, Poland. The study was conducted in this setting due to the convenience of access to participants and the ability to closely monitor survey distribution and completion. Although this may have led to a sample with heightened awareness of infectious diseases, it also ensured higher response rates and engagement from parents directly involved in pediatric care. Parents completed the questionnaires during their child’s hospital stay. Participants provided informed consent before beginning the survey. Responses were collected anonymously to ensure confidentiality. Before statistical analysis, we removed surveys that were illegible or where the majority of answers were missing.

We surveyed 540 individuals, including 414 females and 122 males (3 missing values). The majority of the respondents were between 25 and 34 years old (233, 43.1%) or 35 and 44 years old (212, 39.3%). Of these, 334 (61.6%) had 2 children or more, 264 (48.9%) lived in villages or small towns, and 311 (57.6%) had higher education. Three hundred and twenty participants (59.3%) rated their material status as average, while 152 (28.1%) described it as rather wealthy.

### 2.6. Data Analysis

Data were analyzed using both descriptive and inferential statistics to understand the distribution of responses and identify significant associations and predictors.

Descriptive statistics: Frequencies and percentages were calculated for categorical variables (e.g., gender, education level), while means and standard deviations were computed for continuous variables (e.g., age).

Comparative analysis: Respondents with a generally positive approach towards vaccines were compared with those having a neutral or negative attitude. Chi-squared tests were used to compare categorical variables across groups. For continuous variables, t-tests were employed to determine significant differences.

Multivariate logistic regression: In investigating the determinants of positive attitudes towards LD vaccination, we conducted a multivariate logistic regression analysis utilizing a binary outcome variable—the incidence of a positive attitude towards future LD vaccination, defined as a declaration that one would be somewhat or definitely willing to get vaccinated or vaccinate a child. First, we identified variables associated with a declaration to receive an LD vaccine through a univariate analysis, starting with 24 proposed variables (questions 1–13, and 19–29 related to demographics, experience and knowledge, risk perception and fear, and vaccine attitudes and intentions). Predictor variables, including perceived risk of LD and vaccine safety concerns, were measured using five-point Likert scales, as described in Section 2.3. Demographic factors such as age and education were treated as ordinal variables in the regression analysis. Next, we employed a stepwise backward elimination algorithm. From the initial pool, 4 variables emerged as significant predictors. We calculated the odds ratios and 95% confidence intervals for each of these predictors, offering insights into their relative influence on positive attitudes toward vaccination. We assessed the robustness of our final model by comparing the pseudo-R-squared value of the complete set of variables with that of the reduced model to ensure minimal loss of explanatory power. Additionally, we sought to validate the predictive accuracy of our model by applying cross-validation methods to mitigate overfitting and assess the model’s generalizability.

Handling missing data: Missing data were managed using multiple imputation techniques to ensure robustness in the results. Sensitivity analyses were performed to check the consistency of findings.

Statistical software: Statistical analyses were conducted using R version 4.3.1 (R Core Team, 2023) on Windows 10 Pro 64-bit. The following packages were used for data management and analysis: *rio*, *sjPlot*, *parameters*, *performance*, *ggstatsplot*, *gtsummary*, *MASS*, *readxl*, and *dplyr*.

### 2.7. Ethical Considerations

The study protocol was reviewed and approved by the Bioethical Committee of the Medical University of Bialystok (decision number APK.002.24.2023, 19 January 2023). Informed consent was obtained from all participants, and the study was conducted in accordance with the Declaration of Helsinki. Participants were informed about the purpose of the study, the voluntary nature of their participation, and their right to withdraw at any time without penalty.

## 3. Results

### 3.1. Participant Demographics

Thirty-seven questionnaires were removed due to incomplete data. A total of 503 participants were included. The demographic characteristics and general vaccine attitudes of respondents are summarized in Table S1 (Supplementary SA). In brief, the majority of participants were female (76.1%), aged 25–44, and had higher education (58.6%). Further descriptive insights are provided in Tables S1 and S2 (Supplementaries SA and SB).

### 3.2. General Attitudes Towards Vaccination

**Figure 1:**
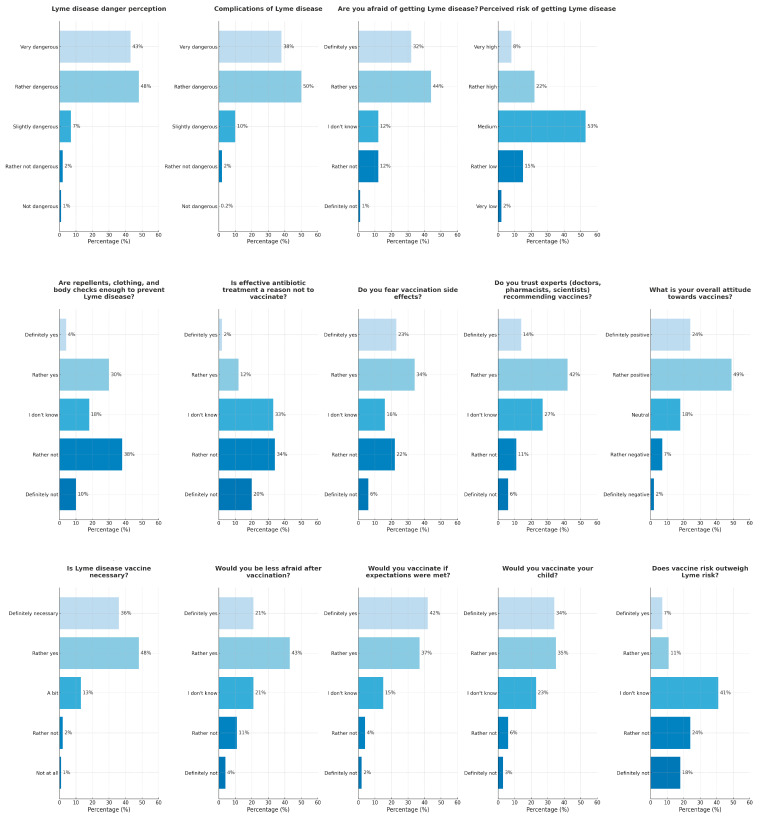
Public perception and attitudes regarding Lyme disease prevention, treatment, and vaccination (*n* = 503). The figure illustrates participants’ views on the perceived danger of Lyme disease and its complications, preventive measures, fear and trust related to vaccination, as well as the perceived necessity and acceptance of a Lyme disease vaccine. The results are divided into three sections: awareness and risk perception of Lyme disease (**first row**), attitudes toward prevention, treatment, and trust in vaccination (**second row**), and intentions regarding Lyme vaccination (**third row**). Percentages reflect the distribution of responses to each survey question.

Public perception and attitudes regarding Lyme disease prevention, treatment, and vaccination are presented in Figure 1. Briefly, within the sample, 364 individuals (72.4%) exhibited a positive attitude towards vaccination in general. The positive vaccination attitude profile was characterized by higher education, residence in larger urban areas, and larger family size (See Supplementary Materials).

More than half of the overall sample (56.46%) expressed definite or moderate concern about vaccine side effects, with those holding negative or neutral attitudes more frequently expressing concern (69.78%) compared to their pro-vaccination counterparts (51.37%), a difference that is statistically significant (*p* < 0.001).

Trust in health experts who recommend vaccination is a significant predictor of vaccination acceptance. A majority of respondents with positive attitudes towards vaccination (70.88%) definitely or rather trust experts, whereas only a minority of those with negative or neutral attitudes share this trust (16.55%), revealing a significant divide (*p* < 0.001). The data also indicate a substantial level of distrust or uncertainty among individuals with negative or neutral attitudes, with 41.01% definitely or rather not trusting experts and 42.45% unsure. Respondents who trust healthcare authorities were much more likely to express a positive attitude towards vaccines (OR 22.84; 95% CI, 12.26–42.52, *p* < 0.001).

### 3.3. Perspectives on LD

#### 3.3.1. Tick Bite Incidences and Perceived Risk

A tick bite in the past was reported by 56.26% of respondents and 51.68% rated their risk of being bitten by a tick as rather high or very high. A history of LD in oneself or a close relative was reported by as many as 202 (40.16%), and 108 (53.46%) of them experienced complications from the disease.

Overall, 90.85% of participants viewed LD as dangerous or very dangerous and 88.27% regarded the complications of LD as dangerous or very dangerous. A fear of contracting LD was expressed by 75.35% of participants, and 30.02% of participants considered their risk of contracting LD as high or very high.

#### 3.3.2. Prevention Methods and Impact of LD Vaccination on Concern Levels

A total of 34.0% (171 respondents) found the use of methods such as repellents, proper clothing, and thorough body checks after outdoor activities to be enough to prevent LD, while 47.7% (240 respondents) considered these measures inadequate. Regarding the belief that effective antibiotic treatments could justify forgoing vaccination, only 13.5% (68 respondents) supported this view. A majority of 53.3% (268 respondents) opposed it. Additionally, 64.21% of participants believed that vaccination would reduce their level of concern about LD.

### 3.4. Predictors of Positive Vaccination Attitudes

**Figure 2:**
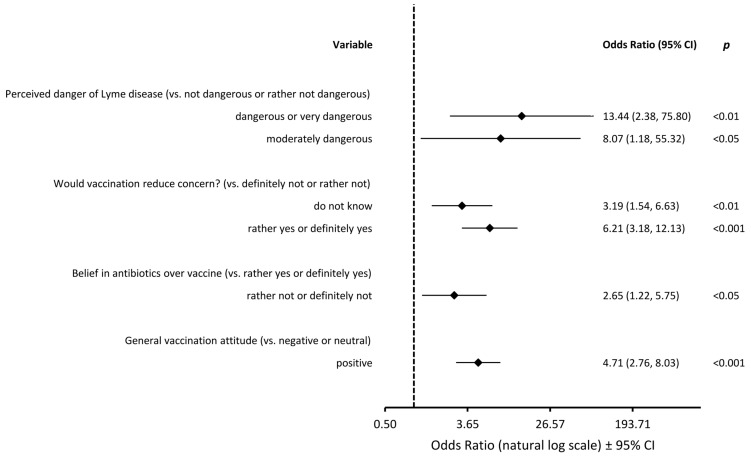
Factors influencing willingness to receive an LD vaccine.

The perception of the necessity for an LD vaccine was overwhelmingly positive, with 84.1% (423 respondents) considering it necessary. Only 12.9% (65 respondents) found it moderately necessary, and a small fraction, 3.0% (15 respondents), deemed it unnecessary. Similarly, the majority think that body checks, repellents, and antibiotics are not sufficient protection against LD and its complications (Figure 2). As many as 412 (81.90%) respondents declared that they would vaccinate themselves or their child if a vaccine that meets their expectations was available (responses “rather yes” and “definitely yes”). Of these 412, 69 (16.74%) wanted to receive this vaccine but declared that they would not vaccinate their child. Sixteen (3.88%) respondents would vaccinate their child only. A negative stance towards LD immunizations (responses “definitely not” and “rather not”) was presented by a minority of respondents. Only 30 (5.96%) respondents would not vaccinate themselves and 45 (8.94%) would not vaccinate their child. The remaining 77 (15.30%) and 115 (22.86%) were unsure (response “do not know”) regarding whether they or their child should receive immunization, respectively.

A multivariate logistic regression analysis identified several significant predictors of positive attitudes towards LD vaccination, defined as willingness to receive a vaccine or to vaccinate a child (Figure 2). Parents with a generally positive attitude towards vaccines, who considered LD as dangerous and believed that a vaccine would reduce their fear of contracting LD, and who also were not certain about the efficacy of antibiotic treatment in LD were more likely to hold favorable views. Sex, place of residence, education level, and number of children had no effect on the odds of willingness to receive an LD vaccine. Similarly, a tick bite in the past, a history of LD, or a history of LD complications in a respondent or her/his close relative were not important predictors of attitude towards an LD vaccine.

This forest plot presents the results of a multivariable logistic regression. Odds ratios (OR) with 95% confidence intervals (CI) are shown, with values greater than 1 indicating a higher likelihood of vaccine acceptance.

### 3.5. Important Aspects Concerning a Future LD Vaccine

**Figure 3:**
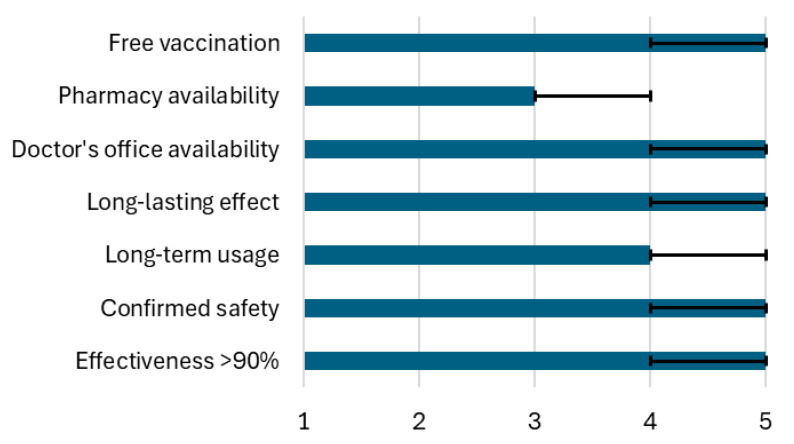
Important aspects of a future LD vaccine measured using a 5-point Likert scale, from 1—unimportant to 5—very important. Data presented as medians (bars) and interquartile range (whiskers).

**Figure 4:**
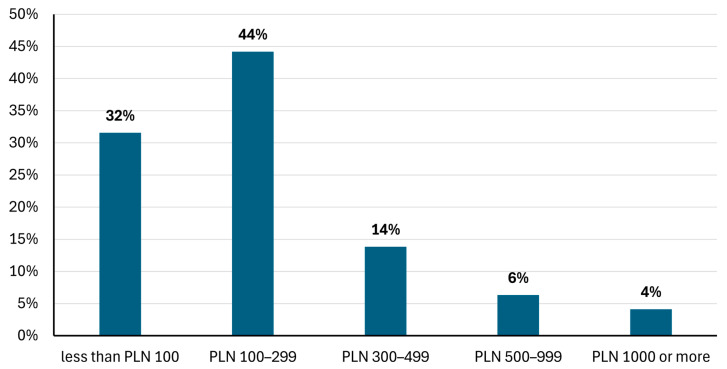
Acceptable vaccine cost per full immunization schedule, declared by respondents with a positive attitude towards a future LD vaccine (n = 412).

Important aspects of a future LD vaccine were analyzed in a group of respondents with a positive attitude toward it (*n* = 412). A majority, 95.14% (392 respondents), rated confirmed vaccination effectiveness of at least 90% as important or very important (Figure 3). Safety confirmed by research was deemed important or very important by 96.35% (397 respondents). Long-term experience with the vaccine and a long-lasting effect without the need for booster doses were valued equally by 88.34% (364 respondents). Vaccine availability at a doctor’s office was crucial for 89.08%, but availability at a pharmacy was considered important for 44.9% of respondents. National Health Fund financing providing free vaccination was important for 86.41% of the respondents. If the vaccine was not funded by the government, the majority of respondents (75%) would be ready to pay no more than PLN 300 per full course of immunization, including all required doses (Figure 4).

### 3.6. Neutral Vaccine Attitudes

The survey data highlight a stark contrast in the perception of vaccine-associated risks versus the risks of LD contraction. A total of 211 respondents (41.9%) believe that the risk associated with an LD vaccine is lower than the risk associated with LD. A minority of the overall sample (17.3%) believe that the risks associated with vaccination definitely or rather outweigh the risks of contracting LD. This belief is more prevalent among those with negative or neutral attitudes towards vaccination (28.78%) in general compared to those with positive attitudes (12.91%), with the difference being statistically significant (*p* < 0.001). Conversely, a higher proportion of pro-vaccination parents (51.37%) either definitely or moderately do not believe that vaccination risks outweigh LD risks, in contrast to only 17.27% of those with negative or neutral attitudes, indicating a substantial discrepancy in perceived risk assessment between the two groups. A substantial proportion of respondents remain uncertain, with 40.8% indicating ‘don’t know’, and a higher prevalence of indecision is observable among those with negative or neutral attitudes towards vaccination (53.96%).

Compared with respondents who believe that vaccination risk does not outweigh LD risk (41.9%), those who are uncertain about LD vaccine risk (40.8%) hold a less positive attitude towards vaccines in general, have less trust in healthcare experts, are more afraid of vaccine side effects, are less likely to accept a future LD vaccine, believe in antibiotics and preventive measures as effective in preventing LD, and rate their risk of getting a tick bite as lower (Supplementary SG). Also, the “I do not know” group tends to be younger, less educated, and more likely to reside in smaller towns or cities. This indicates potential areas which might be addressed in future vaccine campaigns.

A detailed breakdown of the survey responses, both for the overall sample and within the stratified subsets based on parental attitudes towards immunization, can be found in the Supplementary Materials. Additionally, a correlation matrix examining the relationships between the questionnaire responses is available in Supplementary SC and Supplementary Figure S1. Briefly, correlation analysis found an important positive correlation between trust in experts who recommend vaccination and a generally positive attitude towards vaccination (R = 0.6, *p* < 0.001). Higher risk associated with vaccines correlated with lower trust in healthcare experts (R = − 0.41, *p* < 0.001). There was also a negative correlation between belief in the sufficiency of antibiotics as a treatment for Lyme disease and a lower perceived necessity for a vaccine (R = − 0.42; *p* < 0.001).

### 3.7. Unexpected Trends in Vaccine Acceptance

The multivariate analysis did not reveal any significant associations between previous experiences with tick bites or LD and acceptance of an LD vaccine. To further investigate this relationship, we conducted an additional analysis focusing on three subgroups: respondents who had experienced a tick bite (“yes”, *n* = 283 vs. “no”, *n* = 163), those with a personal or familial history of LD (*n* = 202 vs. *n* = 234), and individuals who either experienced LD complications themselves or had close relatives with LD complications (*n* = 111 vs. *n* = 135). For this analysis, we included only respondents who answered “Yes” or “No,” excluding those who selected “I do not know” to ensure clearer results and reduce ambiguity.

Our analysis revealed that a history of LD or its complications was associated with a higher perceived risk of contracting LD and a greater perceived need for an LD vaccine. However, this history did not translate into a higher likelihood of declaring an intention to receive the vaccine.

Similarly, individuals with a history of tick bites expressed a stronger perceived need for an LD vaccine. In contrast to those with a history of LD, respondents who had experienced a tick bite were more likely to state their intention to receive the vaccine.

Additionally, we compared respondents with a tick bite history who had a history of LD (*n* = 140) with those bitten by a tick who did not have a history of LD or its complications (*n* = 143). As previously, we found no associations with an intention to receive an LD vaccine in this group.

We also found that prior experience matters. Individuals with personal or family experiences of tick bites, LD, or LD complications consistently rated the risk of LD higher. Those without prior experience place more faith in preventive measures, while those with prior experience are more skeptical.

Detailed results of this analysis are provided in Supplementaries SD–SF.

## 4. Discussion

Our study identifies key factors that could affect people’s willingness to accept a future LD vaccine. We found that people’s perception of their risk of contracting the disease and their general attitude toward vaccines are the main factors affecting their decision to get vaccinated. Respondents who consider LD as a serious health threat and believe that vaccination can reduce their concerns are more likely to express a willingness to get the vaccine. In contrast, a study by Hook et al. [19] showed that people with a low perceived risk of LD were less interested in vaccination. Our study showed that confidence in healthcare professionals also plays a crucial role, indicating that public campaigns should focus on building trust and addressing parental concerns to improve attitudes toward an LD vaccine. Other research, such as studies by Gould et al. [21] and Stark et al. [22], shows that recommendations from healthcare providers are one of the strongest motivators for vaccine uptake, including an LD vaccine. The topic of trust in medical science in the context of vaccine hesitancy was widely studied by Simione et al. during the COVID-19 pandemic [23]. The authors highlighted the role of psychological aspects, belief in conspiracy theories, and mistrust in science, which influence the decision to receive new vaccines. Simione et al. also designed and validated a test measuring implicit and explicit attitudes toward vaccination [24]. Their instrument appears to be a sensitive and reliable method for measuring the influence of psychological factors, such as conspiracy beliefs and trust in science, that affect the decision-making process. Understanding these psychological aspects in reference to a new Lyme disease vaccine may also be useful in increasing its acceptance, as well as in future studies examining the acceptance of future vaccines.

People who already support vaccines are more likely to adopt new ones. Educating the public about vaccines in general could increase support for LD vaccination. Financial concerns are another important factor. Economic policies, such as providing financial support or coverage, can significantly boost vaccine acceptance. Our previous study found that when vaccines require out-of-pocket payments, their uptake remains low, even after many years on the market [25], emphasizing the need for financial support to make vaccines widely accessible.

Interestingly, many of the participants are skeptical about the effectiveness of current prevention methods, such as using repellents and body checks, viewing them as not good enough for preventing LD. For this reason, LD vaccine acceptance may be further improved by highlighting the role of the vaccine as a supplement to existing non-specific methods of protection against tick bites. Our nuanced findings, however, highlight the importance of addressing younger and less educated populations. This group’s lower trust in experts and reliance on antibiotic treatments suggests that targeted educational campaigns could play a critical role in improving vaccine uptake. Stark et al. mention that non-specific methods are insufficiently effective at reducing LD incidence and underline the need for providing specific preventives such as vaccines [22]. The second interesting finding was skepticism regarding the sufficiency of antibiotic treatments as a reason for not getting vaccinated. Only a small minority of respondents held the view that antibiotics could replace vaccination, which suggests the majority recognize the value of vaccination in preventing LD, rather than relying on treatment after infection. This is a promising result for future vaccination programs, illustrating public understanding of the importance of prevention rather than treatment.

The survey also concentrates on several aspects parents consider important for a future LD vaccine, of which the most essential are vaccine efficacy, safety, long-term use, and easy access. Addressing these concerns could help increase acceptance among those hesitant about vaccination. Our study, like Gidengil et al. [26] and Hook et al. [19], asks questions about a hypothetical vaccine. The willingness to vaccinate may change as more details about the vaccine become available, like estimated cost and number of doses or boosters.

This study has some limitations. The survey targeted the parents of hospitalized children in one single hospital in Poland, which may limit the applicability to other populations or regions. Additionally, the study was conducted in an endemic area for LD; consequently, the results may not apply to regions with different healthcare systems or disease prevalence. Moreover, the exclusion of single adults, seniors, and childless individuals also narrows the captured perspectives on LD vaccination. The sample was heavily skewed toward women and individuals from medium to large urban areas, potentially introducing selection bias and limiting the generalizability of the findings to rural populations and men, who may have different attitudes regarding vaccination. Furthermore, this is a questionnaire-based study that focuses on respondents’ answers in the survey. It does not take into account implicit attitudes or other psychological variables associated with vaccine hesitancy, which are difficult to measure using questionnaires. Moreover, parents completing the survey in a hospital setting may have felt pressure to provide socially desirable answers, particularly regarding trust in health experts and willingness to vaccinate. Finally, focusing on parents of hospitalized children introduces potential bias, as these individuals may be more health-conscious or more aware of vaccination campaigns than the general population.

In conclusion, the results of our study show that LD is perceived as a serious health threat and this perception was a key factor in the acceptance of a future LD vaccine. Awareness of the severity of the disease, as well as fear of contracting LD and its potential complications, were the strongest motivating factors for vaccination. The positive approach was additionally supported by a general acceptance of immunization, which was shown to be greatly influenced by trust in health authorities. Our conclusions highlight the need to build public trust, address concerns related to safety issues, and act decisively on vaccine hesitancy. The vaccination rates of future LD vaccines depend on efforts to increase public awareness about LD, reduce financial barriers, and use the authority of healthcare professionals.

## Data Availability

Data available on reasonable request from the corresponding author.

## Supplementary Materials

The following supporting information can be downloaded at https://www.mdpi.com/article/10.3390/vaccines13010055/s1, Table S1: Sociodemographic characteristics of the study cohort by overall sample and general vaccination attitudes; Table S2: Distribution of survey responses on Lyme disease vaccination in the overall sample and stratified by parental attitudes towards immunization, Table S3: Tick bites in the past and attitudes towards Lyme disease and LD vaccine, Table S4: History of LD and attitudes towards Lyme disease and LD vaccine, Table S5: Complications of Lyme disease in a respondent or close relative and attitudes towards Lyme disease and LD vaccine, Table S6: Comparison of Perceptions, Attitudes, and Demographics Across Respondents Based on Beliefs About Vaccine Risks Relative to Lyme Disease Risk, Figure S1: Spearman’s correlation matrix depicting the relationships between the raw responses to a survey on parental attitudes toward Lyme disease vaccination
