# Supplementary material for: Barriers and Predictors of Lyme Disease Vaccine Acceptance: A Cross-Sectional Study in Poland
Source: Vaccines (Basel). 2025 Jan 10;13(1):55. doi: 10.3390/vaccines13010055 (PMC11769240; doi:10.3390/vaccines13010055)
Supplement: Supplementary file 1 [file vaccines-13-00055-s001.zip › vaccines-3382128-supplementary.pdf]

Content

Supplementaries .... **Error! Bookmark not defined.**

Supplementary SA. Sociodemographic characteristics of the study cohort by overall sample and general vaccination attitudes 2

Supplementary SB. Distribution of survey responses on Lyme disease vaccination in the overall sample and stratified by parental attitudes towards immunization 4

Supplementary SC. Examining the relationships between the questionnaire responses...... 11

Correlation matrix 11

Strong correlations 12

Moderate correlations 12

Weak to moderate correlations 13

Inverse correlations 13

Conclusions 14

Supplementary SD. Tick bites in the past and attitudes towards Lyme disease and LD vaccine.. 15

Supplementary SE. History of LD and attitudes towards Lyme disease and LD vaccine ... 18

Supplementary SF. Complications of Lyme disease in a respondent or close relative and attitudes towards Lyme disease and LD vaccine.. 21

Supplementary SG. Comparison of Perceptions, Attitudes, and Demographics Across Respondents Based on Beliefs About Vaccine Risks Relative to Lyme Disease Risk......... 24

Supplementary SH. Translated survey questions 28

Supplementaries

Supplementary SA. Sociodemographic characteristics of the study cohort by overall sample and general vaccination attitudes

Supplementary Table S1. Sociodemographic characteristics of the study cohort by overall sample and general vaccination attitudes.

Characteristic	*N*	*Overall sample*[a]	*General attitude towards vaccinations*<br>*positive*[a], *N = 364*	 *negative or neutral*[a], *N = 139*	*p*[b]
Sex:	503				0.110
Female		383 (76.14%)	284 (78.02%)	99 (71.22%)	
Male		120 (23.86%)	80 (21.98%)	40 (28.78%)	
Age:	503				
18-24 years		27 (5.37%)	12 (3.30%)	15 (10.79%)	**< 0.01**[d]
25-34 years		218 (43.34%)	155 (42.58%)	63 (45.32%)	0.650[d]
35-44 years		202 (40.16%)	151 (41.48%)	51 (36.69%)	0.380[d]
45 years or above		56 (11.13%)	46 (12.64%)	10 (7.19%)	0.115[d]
Number of children:	503				**< 0.05**
One		173 (34.39%)	115 (31.59%)	58 (41.73%)	
two or above		330 (65.61%	249 (68.41%)	81 (58.27%)	
Place of residence:	503				
Village		141 (28.03%)	100 (27.46%)	41 (29.50%)	0.771[d]
towns and small cities (up to 100,000 inhabitants)		163 (32.41%)	103 (28.29%)	60 (43.16%)	**< 0.01**[d]
medium and large cities (over 100,000 inhabitants)		199 (39.56%)	161 (44.25%)	38 (27.34%)	**< 0.01**[d]
Education:	503				
Primary		12 (2.39%)	8 (2.20%)	4 (2.88%)	0.904[d]
Vocational		37 (7.36%)	23 (6.32%)	14 (10.07%)	0.211[d]
Secondary		159 (31.61%)	94 (25.82%)	65 (46.76%)	**< 0.001**[d]
Higher		295 (58.65%)	239 (65.66%)	56 (40.29%)	**< 0.001**[d]
Financial situation:	503				0.105[c]
wealthy, can afford every expense		19 (3.78%)	11 (3.02%)	8 (5.76%)	

Characteristic	*N*	*Overall sample*[a]	*General attitude towards vaccinations*		*p*[b]
			positive[a], *N = 364*	*negative or neutral*[a], *N = 139*	
quite wealthy, but need to save for bigger expenses		149 (29.62%)	114 (31.32%)	35 (25.18%)	
neither poor nor wealthy		305 (60.64%)	222 (60.99%)	83 (59.71%)	
quite poor, but able to survive		24 (4.77%)	14 (3.85%)	10 (7.19%)	
poor, every expense is a problem		6 (1.19%)	3 (0.82%)	3 (2.16%)	

[a] *n* (%)
[b] Pearson's Chi-squared test
[c] Fisher's exact test
[d] Proportion test

Supplementary SB. Distribution of survey responses on Lyme disease vaccination in the overall sample and stratified by parental attitudes towards immunization

Supplementary Table S2. Distribution of survey responses on Lyme disease vaccination in the overall sample and stratified by parental attitudes towards immunization

Characteristic	*N*	*Overall sample*[a]	*Attitude towards vaccinations* *positive*[a], $n_1 = 364$	*negative or neutral*[a], $n_2 = 139$	p^b
Q1. Interest in the topic of Lyme disease	503				**0.002**
interested or definitely interested		309.00 (61.43%)	239.00 (65.66%)	70.00 (50.36%)	
not interested or neutral		194.00 (38.57%)	125.00 (34.34%)	69.00 (49.64%)	
Q2. The assessment of one's knowledge about the Lyme disease.	503				
low or very low		142.00 (28.23%)	93.00 (25.55%)	49.00 (35.25%)	**0.040**[d]
medium		260.00 (51.69%)	188.00 (51.65%)	72.00 (51.80%)	1.000[d]
high or very high		101.00 (20.08%)	83.00 (22.80%)	18.00 (12.95%)	**0.019**[d]
Q3. Instances of tick bites experienced.	503				0.918
don't know		57.00 (11.33%)	40.00 (10.99%)	17.00 (12.23%)	
no		163.00 (32.41%)	119.00 (32.69%)	44.00 (31.65%)	
yes		283.00 (56.26%)	205.00 (56.32%)	78.00 (56.12%)	
Q4. Perceived risk of being bitten by ticks.	503				
low or very low		51.00 (10.14%)	31.00 (8.52%)	20.00 (14.39%)	0.074[d]
medium		192.00 (38.17%)	128.00 (35.16%)	64.00 (46.04%)	**0.032**[d]
high or very high		260.00 (51.69%)	205.00 (56.32%)	55.00 (39.57%)	**0.001**[d]

Characteristic	*N*	*Overall sample*[a]	*Attitude towards vaccinations* positive[a], $n_1 = 364$	*Attitude towards vaccinations* negative or neutral[a], $n_2 = 139$	p^b
Q5. History of Lyme disease in oneself or among close relatives.	503				0.109
yes		202.00 (40.16%)	145.00 (39.84%)	57.00 (41.01%)	
no		234.00 (46.52%)	177.00 (48.63%)	57.00 (41.01%)	
don't know		67.00 (13.32%)	42.00 (11.54%)	25.00 (17.99%)	
Q6. Occurrence of complications following Lyme disease in oneself or close relatives.	503				0.849
yes		111.00 (22.07%)	78.00 (21.43%)	33.00 (23.74%)	
no		135.00 (26.84%)	99.00 (27.20%)	36.00 (25.90%)	
don't know or not applicable		257.00 (51.09%)	187.00 (51.37%)	70.00 (50.36%)	
Q7. Perceived danger of Lyme disease on a scale from 1 (not dangerous) to 5 (very dangerous).	503				
dangerous or very dangerous		457.00 (90.85%)	341.00 (93.68%)	116.00 (83.45%)	**0.001** [d]
moderately dangerous		35.00 (6.96%)	19.00 (5.22%)	16.00 (11.51%)	**0.022** [d]
harmless or rather harmless		11.00 (2.19%)	4.00 (1.10%)	7.00 (5.04%)	**0.015** [d]
Q8. Perceived severity of complications after Lyme disease.	503				
dangerous or very dangerous		444.00 (88.27%)	332.00 (91.21%)	112.00 (80.58%)	**0.002** [d]
moderately dangerous		48.00 (9.54%)	26.00 (7.14%)	22.00 (15.83%)	**0.005** [d]
harmless or rather harmless		11.00 (2.19%)	6.00 (1.65%)	5.00 (3.60%)	0.082 [d]
Q9. Fear of contracting Lyme disease.	503				
yes or definitely yes		379.00 (75.35%)	288.00 (79.12%)	91.00 (65.47%)	**0.002** [d]
no or definitely no		63.00 (12.52%)	39.00 (10.71%)	24.00 (17.27%)	0.067 [d]

Characteristic	*N*	*Overall sample*[a]	*Attitude towards vaccinations* positive[a], $n_1 = 364$	*Attitude towards vaccinations* negative or neutral[a], $n_2 = 139$	p^b
don't know		61.00 (12.13%)	37.00 (10.16%)	24.00 (17.27%)	**0.042** [d]
Q10. Perceived risk of contracting Lyme disease.	503				0.311
high or very high		151.00 (30.02%)	114.00 (31.32%)	37.00 (26.62%)	
medium		266.00 (52.88%)	193.00 (53.02%)	73.00 (52.52%)	
low or very low		86.00 (17.10%)	57.00 (15.66%)	29.00 (20.86%)	
Q11. Impact of Lyme disease vaccination on the level of concern regarding the disease.	503				
definitely or rather yes		323.00 (64.21%)	266.00 (73.08%)	57.00 (41.01%)	**< 0.001** [d]
definitely or rather no		73.00 (14.51%)	39.00 (10.71%)	34.00 (24.46%)	**< 0.001** [d]
don't know		107.00 (21.27%)	59.00 (16.21%)	48.00 (34.53%)	**< 0.001** [d]
Q12. Adequacy of tick prevention methods, such as the use of repellents, proper clothing, and thorough body checks after outdoor activities, in avoiding Lyme disease.	503				
definitely or rather yes		171.00 (34.00%)	118.00 (32.42%)	53.00 (38.13%)	0.270 [d]
definitely or rather no		240.00 (47.71%)	194.00 (53.30%)	46.00 (33.09%)	**< 0.001** [d]
don't know		92.00 (18.29%)	52.00 (14.29%)	40.00 (28.78%)	**< 0.001** [d]
Q13. Belief in the sufficiency of effective antibiotic treatments for Lyme disease as a reason to forgo vaccination.	503				
definitely or rather yes		68.00 (13.52%)	33.00 (9.07%)	35.00 (25.18%)	**< 0.001** [d]
definitely or rather no		268.00 (53.28%)	230.00 (63.19%)	38.00 (27.34%)	**< 0.001** [d]

Characteristic	*N*	*Overall sample*[a]	*Attitude towards vaccinations* *positive*[a], $n_1 = 364$	*negative or neutral*[a], $n_2 = 139$	p^b
don't know		167.00 (33.20%)	101.00 (27.75%)	66.00 (47.48%)	**< 0.001** [d]
Q14. Perceived necessity for a Lyme disease vaccine.	503				
somewhat or very necessary		423.00 (84.10%)	331.00 (90.93%)	92.00 (66.19%)	**< 0.001** [d]
moderately necessary		65.00 (12.92%)	28.00 (7.69%)	37.00 (26.62%)	**< 0.001** [d]
not at all or not very necessary		15.00 (2.98%)	5.00 (1.37%)	10.00 (7.19%)	**0.002** [d]
Q15. Important aspects concerning a future Lyme disease vaccine:					
15.1. Confirmed vaccination effectiveness of at least 90%.	503				
rather or very important		456.00 (90.66%)	348.00 (95.60%)	108.00 (77.70%)	**< 0.001** [d]
moderately important		35.00 (6.96%)	12.00 (3.30%)	23.00 (16.55%)	**< 0.001** [d]
very or rather unimportant		12.00 (2.39%)	4.00 (1.10%)	8.00 (5.76%)	**0.006** [d]
15.2. Safety of the vaccine confirmed by research.	503				
very or rather important		465.00 (92.45%)	352.00 (96.70%)	113.00 (81.29%)	**< 0.001** [d]
moderately important		31.00 (6.16%)	11.00 (3.02%)	20.00 (14.39%)	**< 0.001** [d]
very or rather unimportant		7.00 (1.39%)	1.00 (0.27%)	6.00 (4.32%)	**0.003** [c]
15.3. Long-term experience with the vaccine's use in the general population.	503				
very or rather important		421.00 (83.70%)	317.00 (87.09%)	104.00 (74.82%)	**0.001** [d]
moderately important		69.00 (13.72%)	40.00 (10.99%)	29.00 (20.86%)	**0.006** [d]

Characteristic	N	Overall sample[a]	Attitude towards vaccinations: positive[a], $n_1 = 364$	Attitude towards vaccinations: negative or neutral[a], $n_2 = 139$	p[b]
very or rather unimportant		13.00 (2.58%)	7.00 (1.92%)	6.00 (4.32%)	0.207 [d]
15.4 Long-lasting effect of the vaccine without the need for booster doses.	503				
very or rather important		419.00 (83.30%)	318.00 (87.36%)	101.00 (72.66%)	**< 0.001** [d]
moderately important		72.00 (14.31%)	43.00 (11.81%)	29.00 (20.86%)	**0.014** [d]
very or rather unimportant		12.00 (2.39%)	3.00 (0.82%)	9.00 (6.47%)	**0.001** [c]
15.5 Vaccine availability at family doctor clinics	503				
very or rather important		414.00 (82.31%)	317.00 (87.09%)	97.00 (69.78%)	**< 0.001** [d]
moderately important		73.00 (14.51%)	40.00 (10.99%)	33.00 (23.74%)	**< 0.001** [d]
very or rather unimportant		16.00 (3.18%)	7.00 (1.92%)	9.00 (6.47%)	**0.021** [d]
15.6 Option to get vaccinated at a pharmacy.	503				0.490
very or rather important		203.00 (40.36%)	146.00 (40.11%)	57.00 (41.01%)	
moderately important		170.00 (33.80%)	119.00 (32.69%)	51.00 (36.69%)	
very or rather unimportant		130.00 (25.84%)	99.00 (27.20%)	31.00 (22.30%)	
15.7 National Health Fund (NFZ) financing the vaccine (free vaccination).	503				
very or rather important		413.00 (82.11%)	307.00 (84.34%)	106.00 (76.26%)	**0.047** [d]
moderately important		68.00 (13.52%)	47.00 (12.91%)	21.00 (15.11%)	0.618 [d]
very or rather unimportant		22.00 (4.37%)	10.00 (2.75%)	12.00 (8.63%)	**0.008** [d]
Q16. Willingness to get vaccinated if the Lyme disease vaccine meets personal criteria.	503				
definitely or rather yes		396.00 (78.73%)	325.00 (89.29%)	71.00 (51.08%)	**< 0.001** [d]

Characteristic	N	Overall sample[a]	Attitude towards vaccinations: positive[a], $n_1 = 364$	Attitude towards vaccinations: negative or neutral[a], $n_2 = 139$	p[b]
definitely or rather no		30.00 (5.96%)	9.00 (2.47%)	21.00 (15.11%)	**< 0.001** [d]
don't know		77.00 (15.31%)	30.00 (8.24%)	47.00 (33.81%)	**< 0.001** [d]
Q17. Willingness to vaccinate one's child if the Lyme disease vaccine is available for children.	503				
definitely or rather yes		343.00 (68.19%)	288.00 (79.12%)	55.00 (39.57%)	**< 0.001** [d]
definitely or rather no		45.00 (8.95%)	11.00 (3.02%)	34.00 (24.46%)	**< 0.001** [d]
don't know		115.00 (22.86%)	65.00 (17.86%)	50.00 (35.97%)	**< 0.001** [d]
Q18. Maximum amount willing to pay for the full vaccination cycle against Lyme disease.	503				
below 100 PLN		179.00 (35.59%)	107.00 (29.40%)	72.00 (51.80%)	**< 0.001** [d]
100-299 PLN		208.00 (41.35%)	160.00 (43.96%)	48.00 (34.53%)	0.069 [d]
300-499 PLN		71.00 (14.12%)	55.00 (15.11%)	16.00 (11.51%)	0.372 [d]
500-999 PLN		28.00 (5.57%)	26.00 (7.14%)	2.00 (1.44%)	**0.022** [c]
1000 PLN and more		17.00 (3.38%)	16.00 (4.40%)	1.00 (0.72%)	0.052 [c]
Q19. Belief whether the risks associated with vaccination outweigh the risks of contracting Lyme disease.	503				
definitely or rather yes		87.00 (17.30%)	47.00 (12.91%)	40.00 (28.78%)	**< 0.001** [d]
definitely or rather no		211.00 (41.95%)	187.00 (51.37%)	24.00 (17.27%)	**< 0.001** [d]
don't know		205.00 (40.76%)	130.00 (35.71%)	75.00 (53.96%)	**< 0.001** [d]

Characteristic	*N*	*Overall sample*[a]	*Attitude towards vaccinations*		*p*[b]
			positive[a], $n_1 = 364$	*negative or neutral*[a], $n_2 = 139$	
Q20. Needle phobia as a reason for vaccination hesitancy.	503				
definitely or rather yes		38.00 (7.55%)	19.00 (5.22%)	19.00 (13.67%)	**0.023** [d]
definitely or rather no		440.00 (87.48%)	336.00 (92.31%)	104.00 (74.82%)	**< 0.001** [d]
don't know		25.00 (4.97%)	9.00 (2.47%)	16.00 (11.51%)	**< 0.001** [d]
Q21. Concern about vaccine side effects.	503				
definitely or rather yes		284.00 (56.46%)	187.00 (51.37%)	97.00 (69.78%)	**< 0.001** [d]
definitely or rather no		139.00 (27.63%)	123.00 (33.79%)	16.00 (11.51%)	**< 0.001** [d]
don't know		80.00 (15.90%)	54.00 (14.84%)	26.00 (18.71%)	0.355 [d]
Q22. Trust in experts (doctors, pharmacists, scientists) who recommend vaccinations.	503				
definitely or rather yes		281.00 (55.86%)	258.00 (70.88%)	23.00 (16.55%)	**< 0.001** [d]
definitely or rather no		85.00 (16.90%)	28.00 (7.69%)	57.00 (41.01%)	**< 0.001** [d]
don't know		137.00 (27.24%)	78.00 (21.43%)	59.00 (42.45%)	**< 0.001** [d]

[a] *n* (%)
[b] Pearson’s Chi-squared test
[c] Fisher’s exact test
[d] Proportion test

Supplementary SC. Examining the relationships between the questionnaire responses

Correlation matrix

In order to understand the complex web of factors that influence parental decision-making regarding Lyme disease vaccination, a comprehensive survey was conducted. Raw data derived from 3-point and 5-point Likert scales were subjected to analysis using Spearman's rank correlation coefficient to examine the underlying interrelationships. The following correlation matrix in Figure S1 presents a visual representation of these relationships, offering insights into how various perceptions and attitudes are interconnected.

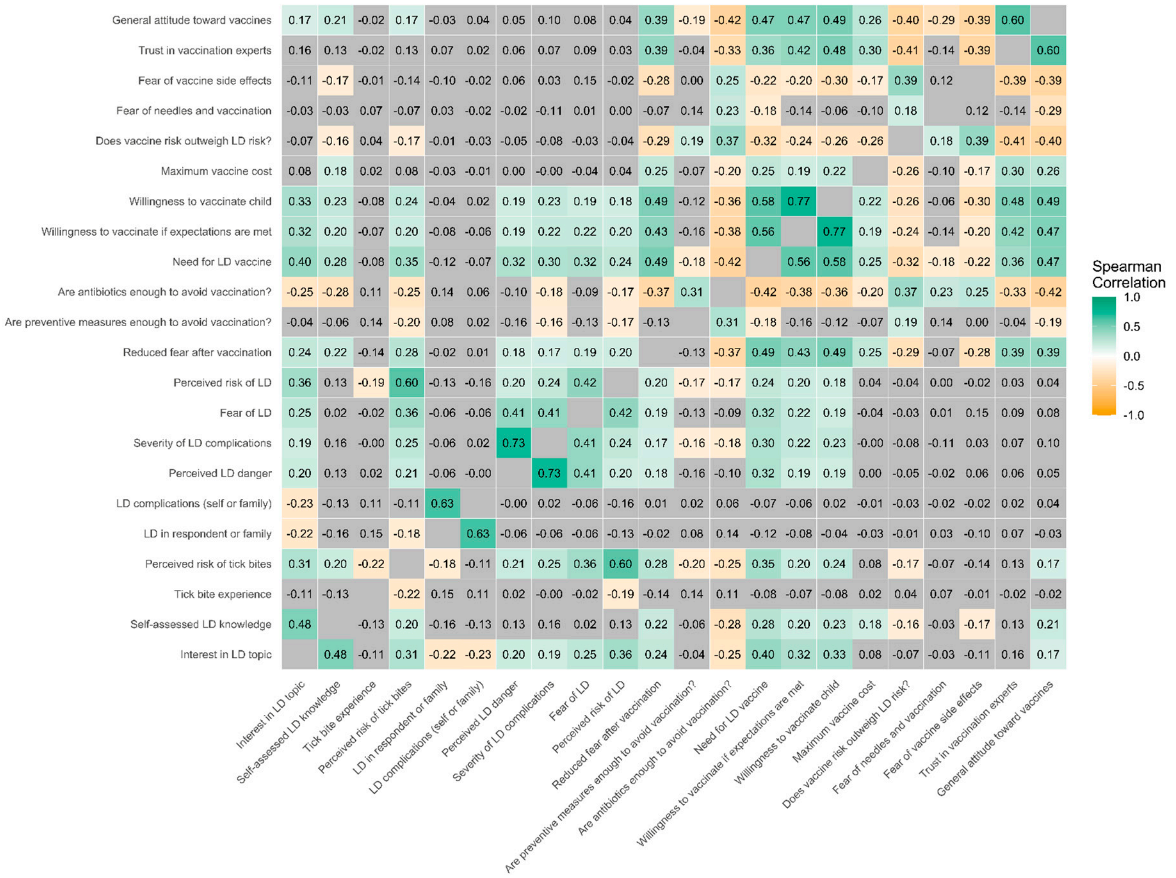

Figure S1. Spearman's correlation matrix depicting the relationships between the raw responses to a survey on parental attitudes toward Lyme disease vaccination.

Based on the data from the Figure S1, several analytical conclusions can be drawn.

Strong correlations

Perceived danger and severity (Q7 vs. Q8)

The strong positive correlation between the perceived danger of Lyme disease and the perceived severity of complications after the disease suggests that individuals who consider Lyme disease to be dangerous are also more likely to view its complications as severe. This relationship underscores the fear and concern associated with the disease and its long-term impacts.

Willingness to vaccinate oneself and child (Q16 vs. Q17)

There is also a strong positive correlation between the willingness to get vaccinated and the willingness to vaccinate one's child. This suggests that personal health decisions strongly reflect parental attitudes towards vaccination for their children, indicating a consistency in vaccine acceptance across different age groups.

Moderate correlations

History of Lyme disease and complications (Q5 vs. Q6).

A moderate positive correlation between one's history of Lyme disease and the occurrence of complications in oneself or close relatives indicates that personal experience with the disease and its more severe outcomes are linked. This could drive greater awareness and concern, potentially influencing attitudes towards prevention.

Perceived tick bite risk and Lyme disease risk (Q4 vs. Q10)

The correlation here suggests that individuals who perceive a higher risk of being bitten by ticks also perceive a higher risk of contracting Lyme disease. This indicates an understanding of the relationship between tick exposure and disease risk.

Trust in experts and general attitude towards vaccinations (Q22 vs. Q23)

A moderate positive correlation between trust in experts who recommend vaccinations and a general positive attitude towards vaccinations implies that expert opinion can be influential in shaping overall vaccine sentiment.

Weak to moderate correlations

Interest in Lyme disease and perceived necessity for vaccine (Q1 vs. Q14)

Individuals who have an interest in Lyme disease are somewhat more likely to perceive the necessity for a Lyme disease vaccine. This suggests that engagement with the topic can influence perceived vaccine importance.

Impact of vaccination on concern and willingness to vaccinate child (Q11 vs. Q17)

The correlation here suggests that those who believe that vaccination would reduce their level of concern about Lyme disease might also be more willing to vaccinate their children, reflecting consistency in perceived vaccine benefits across different family members.

Perceived necessity for vaccine and willingness to get vaccinated (Q14 vs. Q16)

A moderate positive correlation indicates that seeing a Lyme disease vaccine as necessary is associated with a higher willingness to get vaccinated, which is intuitive as perceived need would drive vaccination intentions.

Inverse correlations

Impact of vaccination on concern and belief in vaccination risks (Q11 vs. Q19)

The inverse correlation indicates that those who think vaccination will reduce their concern about Lyme disease are less likely to believe that the risks associated with vaccination outweigh the risks of contracting the disease.

Sufficiency of antibiotic treatments and perceived necessity for vaccine (Q13 vs. Q14)

This negative correlation suggests that belief in the sufficiency of antibiotics as a treatment for Lyme disease is associated with a lower perceived necessity for a vaccine. This implies a potential barrier to vaccine uptake due to the belief in alternative treatments.

Belief in vaccination risks and trust in experts (Q19 vs. Q22)

The negative correlation here indicates that those who are more worried about the risks associated with vaccination are less likely to trust experts who recommend vaccinations. This points to the role of trust in mitigating fears about vaccination.

Conclusions

These observations can inform public health strategies for increasing Lyme disease vaccine uptake. For instance, emphasizing vaccine safety and efficacy, leveraging the trust in healthcare professionals, and addressing concerns about vaccine risks are likely to be effective. Moreover, experiences with Lyme disease and its complications, as well as perceptions of risk and severity, are important factors influencing attitudes toward vaccination and should be carefully addressed in educational campaigns.

Supplementary SD. Tick bites in the past and attitudes towards Lyme disease and LD vaccine

Supplementary Table S3. Tick bites in the past and attitudes towards Lyme disease and LD vaccine

Question	Bitten by a tick in the past			Not bitten by a tick in the past			
	n	Mean, SD	Median, IQR	n	Mean, SD	Median, IQR	p
4. What do you think is your risk of being bitten by a tick?	283	3.82 ±0.93	4 (3-5)	163	3.233 ±0.798	3 (3-4)	<0.001
7. On a scale from 1 to 5, how dangerous do you think Lyme disease is? (1 = Not dangerous, 5 = Very dangerous)	283	4.311 ±0.675	4 (4-5)	163	4.288 ±0.791	4 (4-5)	0.823
8. How serious do you believe the complications after having Lyme disease are?	283	4.261 ±0.681	4 (4-5)	163	4.184 ±0.803	4 (4-5)	0.586
9. Are you afraid of contracting Lyme disease?	283	3.947 ±1.028	4 (4-5)	163	3.883 ±0.977	4 (3-5)	0.357
10. What do you think is your risk of contracting Lyme disease?	283	3.357 ±0.877	3 (3-4)	163	2.853 ±0.739	3 (2-3)	<0.001
11. If you were vaccinated against Lyme disease, would you be less afraid of this disease?	283	3.82 ±0.978	4 (3-5)	163	3.472 ±1.146	4 (3-4)	0.004
12. Do you believe that protective measures such as using tick repellents, wearing appropriate clothing, and carefully checking your body after outdoor activities are enough to prevent Lyme disease?	283	2.661 ±1.11	2 (2-4)	163	3.012 ±1.048	3 (2-4)	0.002
13. Do you think that the availability of effective antibiotic treatments for Lyme disease is a sufficient reason to forgo vaccination?	283	2.314 ±0.998	2 (2-3)	163	2.613 ±1.002	3 (2-3)	0.003
14. How necessary do you think a vaccine for Lyme disease is?	283	4.237 ±0.77	4 (4-5)	163	4.031 ±0.812	4 (4-5)	0.014

16. Would you be willing to get vaccinated if the Lyme disease vaccine met your requirements?	283	4.163 ±0.997	4 (4-5)	163	4.018 ±0.926	4 (4-5)	0.043
17. Would you vaccinate your child if a Lyme disease vaccine was available for children?	283	3.961 ±1.07	4 (3-5)	163	3.748 ±1.044	4 (3-5)	0.023
19. Do you believe that the risks associated with vaccination are greater than the risks of contracting Lyme disease?	283	2.604 ±1.154	3 (2-3)	163	2.742 ±1.028	3 (2-3)	0.194
20. Is fear of needles a reason why you are hesitant about vaccinations?	283	1.555 ±1	1 (1-2)	163	1.571 ±0.962	1 (1-2)	0.479
21. Are you afraid of the side effects of vaccines?	283	3.449 ±1.272	4 (2-4)	163	3.497 ±1.146	4 (2-4)	0.890
22. Do you trust experts (doctors, pharmacists, scientists) who recommend vaccinations?	283	3.484 ±1.106	4 (3-4)	163	3.417 ±0.993	4 (3-4)	0.345
23. What is your general attitude towards vaccinations?	283	3.862 ±0.948	4 (3-5)	163	3.828 ±0.953	4 (3-4)	0.748
24. Gender:	283			163			0.036
Female, n (%)	283	208 (73.5%)			134 (82.2%)		
Male, n (%)	283	75 (26.5%)			29 (17.8%)		
25. Age:	283			163			0.233
18-24 years		11 (3.9%)			13 (8%)		
25-34 years		123 (43.5%)			75 (46%)		
35-44 years		118 (41.7%)			59 (36.2%)		
45 years or above		31 (11%)			16 (9.8%)		
26. How many children do you have?	283			163			0.005
One		79 (27.9%)			70 (42.9%)		0.001
Two		146 (51.6%)			64 (39.3%)		0.012

Three or more		58 (20.5%)	29 (17.8%)	0.488
27. Place of residence:	283		163	0.163
Village		88 (31.1%)	37 (22.7%)	
towns and small cities (up to 100,000 inhabitants)		88 (31.1%)	56 (34.4%)	
medium and large cities (over 100,000 inhabitants)		107 (37.8%)	70 (42.9%)	
28. Education level:	283		163	0.094
Primary		4 (1.4%)	8 (4.9%)	
Vocational		19 (6.7%)	14 (8.6%)	
Secondary		82 (29%)	51 (31.3%)	
Higher		178 (62.9%)	90 (55.2%)	
29. How would you rate your financial situation?	283		163	0.884
wealthy, can afford every expense		14 (4.9%)	3 (1.8%)	0.287
quite wealthy, but need to save for bigger expenses		86 (30.4%)	43 (26.4%)	
neither poor nor wealthy		166 (58.7%)	108 (66.3%)	
quite poor, but able to survive		14 (4.9%)	6 (3.7%)	
poor, every expense is a problem		3 (1.1%)	3 (1.8%)	

Both medians with interquartile ranges (IQR) and means with standard deviations (SD) are presented to provide a comprehensive understanding of the data. Medians and IQRs are reported as the primary measures of central tendency and variability, reflecting the ordinal nature of the Likert scale data and ensuring appropriate interpretation. Means and SDs are included for descriptive purposes to offer additional insight into the distribution and variability of responses, facilitating easier comparisons across groups. The statistical significance was assessed using the non-parametric Mann-Whitney U test, which is appropriate for ordinal data, while means serve as supplementary information to enhance interpretability. Categorical variables in questions 24 through 29 were analyzed using the chi-square test. If the chi-square test indicated significant differences, pairwise comparisons were conducted.

Supplementary SE. History of LD and attitudes towards Lyme disease and LD vaccine

Supplementary Table S4. History of LD and attitudes towards Lyme disease and LD vaccine

Question	History of Lyme disease in oneself or in a close relative			No history of Lyme disease in oneself or in a close relative			
	n	Mean, SD	Median, IQR	n	Mean, SD	Median, IQR	p
4. What do you think is your risk of being bitten by a tick?	202	3.866 ±0.971	4 (3-5)	234	3.363 ±0.829	3 (3-4)	**<0.001**
7. On a scale from 1 to 5, how dangerous do you think Lyme disease is? (1 = Not dangerous, 5 = Very dangerous)	202	4.351 ±0.677	4 (4-5)	234	4.295 ±0.771	4 (4-5)	0.666
8. How serious do you believe the complications after having Lyme disease are?	202	4.292 ±0.712	4 (4-5)	234	4.222 ±0.719	4 (4-5)	0.338
9. Are you afraid of contracting Lyme disease?	202	4.015 ±0.944	4 (4-5)	234	3.885 ±1.048	4 (3-5)	0.309
10. What do you think is your risk of contracting Lyme disease?	202	3.371 ±0.867	3 (3-4)	234	2.991 ±0.849	3 (3-3)	**<0.001**
11. If you were vaccinated against Lyme disease, would you be less afraid of this disease?	202	3.688 ±1.054	4 (3-4)	234	3.662 ±1.069	4 (3-4)	0.809
12. Do you believe that protective measures such as using tick repellents, wearing appropriate clothing, and carefully checking your body after outdoor activities are enough to prevent Lyme disease?	202	2.649 ±1.102	2 (2-4)	234	2.97 ±1.09	3 (2-4)	**0.004**
13. Do you think that the availability of effective antibiotic treatments for Lyme disease is a sufficient reason to forgo vaccination?	202	2.282 ±1.039	2 (1-3)	234	2.487 ±0.995	2 (2-3)	**0.034**
14. How necessary do you think a vaccine for Lyme disease is?	202	4.272 ±0.753	4 (4-5)	234	4.12 ±0.815	4 (4-5)	0.080

16. Would you be willing to get vaccinated if the Lyme disease vaccine met your requirements?	202	4.183 ±0.957	4 (4-5)	234	4.09 ±0.974	4 (4-5)	0.282
17. Would you vaccinate your child if a Lyme disease vaccine was available for children?	202	3.926 ±1.06	4 (3-5)	234	3.88 ±1.054	4 (3-5)	0.634
19. Do you believe that the risks associated with vaccination are greater than the risks of contracting Lyme disease?	202	2.703 ±1.155	3 (2-3)	234	2.585 ±1.09	3 (2-3)	0.448
20. Is fear of needles a reason why you are hesitant about vaccinations?	202	1.584 ±1.02	1 (1-2)	234	1.526 ±0.923	1 (1-2)	0.917
21. Are you afraid of the side effects of vaccines?	202	3.554 ±1.209	4 (3-4)	234	3.47 ±1.219	4 (2-4)	0.475
22. Do you trust experts (doctors, pharmacists, scientists) who recommend vaccinations?	202	3.386 ±1.032	4 (3-4)	234	3.517 ±1.081	4 (3-4)	0.138
23. What is your general attitude towards vaccinations?	202	3.847 ±0.931	4 (3-4)	234	3.897 ±0.948	4 (4-5)	0.451
24. Gender:	202			234			0.294
Female, n (%)		153 (75.7%)			187 (79.9%)		
Male, n (%)		49 (24.35)			47 (20.1%)		
25. Age:	202			234			0.291
18-24 years		6 (3.0%)			13 (5.5%)		
25-34 years		94 (46.5%)			94 (40.2%)		
35-44 years		77 (38.1%)			102 (43.6%)		
45 years or above		25 (12.4%)			25 (10.7%)		
26. How many children do you have?	202			234			0.084
One		56 (27.7%)			88 (37.6%)		
Two		102 (50.5%)			99 (42.3%)		

Three or more		44 (21.8%)		47 (20.1%)	
27. Place of residence:	202		234		
Village		66 (32.7%)		56 (23.9%)	
towns and small cities (up to 100,000 inhabitants)		58 (28.7%)		83 (35.5%)	
medium and large cities (over 100,000 inhabitants)		78 (38.6%)		95 (40.6%)	
28. Education level:	202		234		0.384
Primary		3 (1.5%)		8 (3.4%)	
Vocational		14 (6.9%)		22 (9.4%)	
Secondary		57 (28.2%)		69 (29.5%)	
Higher		128 (63.4%)		135 (57.7%)	
29. How would you rate your financial situation?	202		234		0.884
wealthy, can afford every expense		9 (4.5%)		10 (4.3%)	0.478
quite wealthy, but need to save for bigger expenses		59 (29.2%)		73 (31.2%)	
neither poor nor wealthy		123 (60.9%)		136 (58.1%)	
quite poor, but able to survive		11 (5.4%)		10 (4.3%)	
poor, every expense is a problem		0 (0%)		5 (2.1%)	

Both medians with interquartile ranges (IQR) and means with standard deviations (SD) are presented to provide a comprehensive understanding of the data. Medians and IQRs are reported as the primary measures of central tendency and variability, reflecting the ordinal nature of the Likert scale data and ensuring appropriate interpretation. Means and SDs are included for descriptive purposes to offer additional insight into the distribution and variability of responses, facilitating easier comparisons across groups. The statistical significance was assessed using the non-parametric Mann-Whitney U test, which is appropriate for ordinal data, while means serve as supplementary information to enhance interpretability. Categorical variables in questions 24 through 29 were analyzed using the chi-square test. If the chi-square test indicated significant differences, pairwise comparisons were conducted.

Supplementary SF. Complications of Lyme disease in a respondent or close relative and attitudes towards Lyme disease and LD vaccine

Supplementary Table S5. Complications of Lyme disease in a respondent or close relative and attitudes towards Lyme disease and LD vaccine

Question	Experienced complications of Lyme disease			Did not experience complications of Lyme disease			
	n	Mean, SD	Median, IQR	n	Mean, SD	Median, IQR	p
4. What do you think is your risk of being bitten by a tick?	111	3.829 ±0.971	4 (3-5)	135	3.467 ±0.904	3 (3-4)	0.003
7. On a scale from 1 to 5, how dangerous do you think Lyme disease is? (1 = Not dangerous, 5 = Very dangerous)	111	4.45 ±0.599	5 (4-5)	135	4.148 ±0.824	4 (4-5)	0.009
8. How serious do you believe the complications after having Lyme disease are?	111	4.315 ±0.7	4 (4-5)	135	4.141 ±0.745	4 (4-5)	0.086
9. Are you afraid of contracting Lyme disease?	111	4.144 ±0.84	4 (4-5)	135	3.8 ±1.064	4 (3-5)	0.022
10. What do you think is your risk of contracting Lyme disease?	111	3.441 ±0.891	3 (3-4)	135	3.119 ±0.89	3 (3-4)	0.010
11. If you were vaccinated against Lyme disease, would you be less afraid of this disease?	111	3.757 ±1.105	4 (3-5)	135	3.489 ±1.145	4 (3-4)	0.064
12. Do you believe that protective measures such as using tick repellents, wearing appropriate clothing, and carefully checking your body after outdoor activities are enough to prevent Lyme disease?	111	2.631 ±1.053	2 (2-3)	135	2.963 ±1.109	3 (2-4)	0.024
13. Do you think that the availability of effective antibiotic treatments for Lyme disease is a sufficient reason to forgo vaccination?	111	2.324 ±1.097	2 (1-3)	135	2.422 ±0.996	2 (2-3)	0.334
14. How necessary do you think a vaccine for Lyme disease is?	111	4.351 ±0.746	4 (4-5)	135	4.037 ±0.85	4 (4-5)	0.005

16. Would you be willing to get vaccinated if the Lyme disease vaccine met your requirements?	111	4.243 ±0.946	5 (4-5)	135	3.993 ±1.103	4 (3-5)	0.095
17. Would you vaccinate your child if a Lyme disease vaccine was available for children?	111	3.901 ±1.16	4 (3-5)	135	3.778 ±1.084	4 (3-5)	0.269
19. Do you believe that the risks associated with vaccination are greater than the risks of contracting Lyme disease?	111	2.73 ±1.228	3 (2-3)	135	2.652 ±1.074	3 (2-3)	0.900
20. Is fear of needles a reason why you are hesitant about vaccinations?	111	1.604 ±0.975	1 (1-2)	135	1.578 ±1.011	1 (1-2)	0.901
21. Are you afraid of the side effects of vaccines?	111	3.387 ±1.215	4 (2-4)	135	3.637 ±1.244	4 (3-5)	0.093
22. Do you trust experts (doctors, pharmacists, scientists) who recommend vaccinations?	111	3.495 ±1.017	4 (3-4)	135	3.444 ±1.084	4 (3-4)	0.816
23. What is your general attitude towards vaccinations?	111	3.811 ±0.977	4 (3-4)	135	3.83 ±0.951	4 (3-4)	0.880
24. Gender:	111						0.415
Female, n (%)		84 (75.7%)			108 (80%)		
Male, n (%)		27 (24.3%)			27 (20%)		
25. Age:	111			135			0.483
18-24 years		4 (3.6%)			5 (3.7%)		
25-34 years		52 (46.8%)			53 (39.3%)		
35-44 years		41 (36.9%)			63 (46.7%)		
45 years or above		14 (12.6%)			14 (10.4%)		
26. How many children do you have?	111			135			0.187
One		34 (30.6%)			48 (35.6%)		
Two		57 (51.4%)			54 (40%)		

Three or more		20 (18%)		33 (24.4%)	
27. Place of residence:	111		135		0.766
Village		33 (29.7%)		39 (28.9%)	
towns and small cities (up to 100,000 inhabitants)		40 (36%)		44 (32.6%)	
medium and large cities (over 100,000 inhabitants)		38 (34.2%)		52 (38.5%)	
28. Education level:	111		135		0.417
Primary		2 (1.8%)		3 (2.2%)	
Vocational		9 (8.1%)		16 (11.9%)	
Secondary		26 (23.4%)		40 (29.6%)	
Higher		74 (66.7%)		76 (56.3%)	
29. How would you rate your financial situation?	111		135		0.594
wealthy, can afford every expense		3 (2.7%)		6 (4.4%)	
quite wealthy, but need to save for bigger expenses		34 (30.6%)		34 (25.2%)	
neither poor nor wealthy		67 (60.4%)		86 (63.7%)	
quite poor, but able to survive		7 (6.3%)		6 (4.4%)	
poor, every expense is a problem		0 (0%)		3 (2.2%)	

Both medians with interquartile ranges (IQR) and means with standard deviations (SD) are presented to provide a comprehensive understanding of the data. Medians and IQRs are reported as the primary measures of central tendency and variability, reflecting the ordinal nature of the Likert scale data and ensuring appropriate interpretation. Means and SDs are included for descriptive purposes to offer additional insight into the distribution and variability of responses, facilitating easier comparisons across groups. The statistical significance was assessed using the non-parametric Mann-Whitney U test, which is appropriate for ordinal data, while means serve as supplementary information to enhance interpretability. Categorical variables in questions 24 through 29 were analyzed using the chi-square test. If the chi-square test indicated significant differences, pairwise comparisons were conducted.

Supplementary SG. Comparison of Perceptions, Attitudes, and Demographics Across Respondents Based on Beliefs About Vaccine Risks Relative to Lyme Disease Risk.

Supplementary Table S6. Comparison of Perceptions, Attitudes, and Demographics Across Respondents Based on Beliefs About Vaccine Risks Relative to Lyme Disease Risk

Question	Do you believe that the risks associated with vaccination are greater than the risks of contracting Lyme disease?									
	Yes			No			I do not know			p
	n	Mean, SD	Median, IQR	n	Mean, SD	Median, IQR	n	Mean, SD	Median, IQR	
4. What do you think is your risk of being bitten by a tick?	87	3.471 ±0.998	3 (3-4) ^^	211	3.796 ±0.932	4 (3-5) ##**	205	3.473 ±0.883	3 (3-4) ^^	
7. On a scale from 1 to 5, how dangerous do you think Lyme disease is? (1 = Not dangerous, 5 = Very dangerous)	87	4.253 ±0.824	4 (4-5)	211	4.351 ±0.73	4 (4-5)	205	4.283 ±0.663	4 (4-5) *	
8. How serious do you believe the complications after having Lyme disease are?	87	4.241 ±0.835	4 (4-5)	211	4.322 ±0.717	4 (4-5)	205	4.161 ±0.663	4 (4-5)	
9. Are you afraid of contracting Lyme disease?	87	3.793 ±1.163	4 (3-5)	211	3.976 ±0.963	4 (4-5)	205	3.956 ±0.946	4 (3-5)	
10. What do you think is your risk of contracting Lyme disease?	87	3.126 ±1.043	3 (2-4)	211	3.237 ±0.84	3 (3-4)	205	3.161 ±0.816	3 (3-4)	
11. If you were vaccinated against Lyme disease, would you be less afraid of this disease?	87	3.379 ±1.287	4 (2-4) ^^^	211	4.014 ±0.949	4 (4-5) ###***	205	3.454 ±0.915	4 (3-4) ^^^	
12. Do you believe that protective measures such as using tick repellents, wearing appropriate clothing, and carefully checking your body after outdoor activities are enough to prevent Lyme disease?	87	3.126 ±1.159	3 (2-4) ^^^	211	2.573 ±1.064	2 (2-4) ##***	205	2.902 ±1.039	3 (2-4) ^^	

13. Do you think that the availability of effective antibiotic treatments for Lyme disease is a sufficient reason to forgo vaccination?	87	2.701 ±1.221	3 (2-4) ^^^	211	1.967 ±0.912	2 (1-2)###***	205	2.776 ±0.746	3 (2-3) ^^^	
14. How necessary do you think a vaccine for Lyme disease is?	87	3.885 ±1.016	4 (3-5) ^^^	211	4.445 ±0.669	5 (4-5) ###***	205	4.005 ±0.69	4 (4-4) ^^^	
16. Would you be willing to get vaccinated if the Lyme disease vaccine met your requirements?	87	3.862 ±1.277	4 (3-5) ^^	211	4.379 ±0.861	5 (4-5) ###**	205	3.961 ±0.822	4 (4-5) ^^^	
17. Would you vaccinate your child if a Lyme disease vaccine was available for children?	87	3.598 ±1.41	4 (2-5) ^^	211	4.199 ±0.98	4 (4-5) ###**	205	3.707 ±0.818	4 (3-4) ^^^	
20. Is fear of needles a reason why you are hesitant about vaccinations?	87	1.759 ±1.21	1 (1-2)	211	1.403 ±0.858	1 (1-2)	205	1.678 ±0.987	1 (1-2) ^^	
21. Are you afraid of the side effects of vaccines?	87	3.862 ±1.322	4 (3-5) ^^^	211	2.91 ±1.174	3 (2-4) ###***	205	3.849 ±0.986	4 (3-5) ^^^	
22. Do you trust experts (doctors, pharmacists, scientists) who recommend vaccinations?	87	2.92 ±1.269	3 (2-4) ^^^	211	3.943 ±0.939	4 (4-5) ###***	205	3.215 ±0.842	3 (3-4) ^^^	
23. What is your general attitude towards vaccinations?	87	3.322 ±1.262	4 (2-4) ^^^	211	4.237 ±0.769	4 (4-5) ###***	205	3.678 ±0.743	4 (3-4) ^^^	
24. Gender:	87			211			205			0.854
Female		68 (78.2%)			161 (76.3%)			154 (75.1%)		
Male		19 (21.8%)			50 (23.7%)			51 (24.9%)		
25. Age:										0.04
18-24 years		5 (5.7%)			6 (2.8%)			16 (7.8%)		0.079
25-34 years		33 (37.9%)			84 (39.8%)			101 (49.3%)		0.080

35-44 years	40 (46%)	92 (43.6%)	70 (34.1%)	0.069
45 years or above	9 (10.3%)	29 (13.7%)	18 (8.8%)	0.265
26. How many children do you have?				0.108
One	22 (25.3%)	73 (34.6%)	78 (38%)	
Two	41 (47.1%)	94 (44.5%)	95 (46.3%)	
Three or more	24 (27.6%)	44 (20.9%)	32 (15.6%)	
27. Place of residence:				<0.001
Village	24 (27.6%)	56 (26.5%)	61 (29.8%)	0.543
towns and small cities (up to 100,000 inhabitants)	37 (42.5%)	48 (22.7%)	78 (38%)	<0.001
medium and large cities (over 100,000 inhabitants)	26 (29.9%)	107 (50.7%)	66 (32.2%)	<0.001
28. Education level:				<0.001
Primary	3 (3.4%)	3 (1.4%)	6 (2.9%)	0.467
Vocational	13 (14.9%)	7 (3.3%)	17 (8.3%)	0.002
Secondary	29 (33.3%)	39 (18.5%)	91 (44.4%)	<0.001
Higher	42 (48.3%)	162 (76.8%)	91 (44.4%)	<0.001
29. How would you rate your financial situation?				0.709

wealthy, can afford every expense	3 (3.4%)	9 (4.3%)	7 (3.4%)
quite wealthy, but need to save for bigger expenses	23 (26.4%)	81 (38.4%)	45 (22%)
neither poor nor wealthy	55 (63.2%)	110 (52.1%)	140 (68.3%)
quite poor, but able to survive	6 (6.9%)	7 (3.3%)	11 (5.4%)
poor, every expense is a problem	0 (0%)	4 (1.9%)	2 (1%)

(*) indicates significance vs "Yes",

(#) indicates significant difference when compared with "I do not know"

(^) indicates significant difference when compared with "No"

A single symbol indicates $p<0.05$, a double indicates $p<0.01$, and a triple $p<0.001$

Both medians with interquartile ranges (IQR) and means with standard deviations (SD) are presented to provide a comprehensive understanding of the data. Medians and IQRs are reported as the primary measures of central tendency and variability, reflecting the ordinal nature of the Likert scale data and ensuring appropriate interpretation. Means and SDs are included for descriptive purposes to offer additional insight into the distribution and variability of responses, facilitating easier comparisons across groups. The statistical significance was assessed using the non-parametric multiple comparisons of mean rank test for all groups, which is appropriate for ordinal data, while means serve as supplementary information to enhance interpretability. Categorical variables in questions 24 through 29 were analyzed using the chi-square test. If the chi-square test indicated significant differences, pairwise comparisons were conducted.

Supplementary SH. Translated survey questions

"Survey on Parental Opinions About Lyme Disease Vaccinations"

Tick-borne diseases, such as Lyme disease, are a significant health concern in our region. The purpose of this survey is to understand your opinions on Lyme disease. Completing the survey should take no more than 5 minutes and will certainly contribute to improving the health of the population in our region. The survey is fully anonymous, and the information collected will be used solely for aggregated scientific analysis. You may withdraw from the study at any point. Completing the survey is equivalent to consenting to participate in the study.

1. Are you interested in the topic of Lyme disease?

☐ 1. Definitely not

☐ 2. Rather not

☐ 3. I don't know

☐ 4. Rather yes

☐ 5. Definitely yes

2. How would you rate your knowledge about this disease?

☐ 1. Very low

☐ 2. Rather low

☐ 3. Average

☐ 4. Rather high

☐ 5. Very high

3. Have you ever been bitten by a tick?

☐ 1. Yes

☐ 2. No

☐ 3. I don’t know

4. What do you think is your risk of being bitten by a tick?

☐ 1. Very low

☐ 2. Rather low

☐ 3. Average

☐ 4. Rather high

☐ 5. Very high

5. Have you or anyone close to you ever had Lyme disease?

☐ 1. Yes

☐ 2. No

☐ 3. I don’t know

6. If so, did you or anyone close to you experience complications from this disease?

☐ 1. Yes

☐ 2. No

☐ 3. I don’t know

☐ 4. Not applicable

7. On a scale from 1 to 5, how dangerous do you think Lyme disease is?

(1 = Not dangerous, 5 = Very dangerous)

☐ 1. Not dangerous

☐ 2. Rather not dangerous

☐ 3. Moderately dangerous

☐ 4. Rather dangerous

☐ 5. Very dangerous

8. How serious do you believe the complications after having Lyme disease are?

☐ 1. Not serious

☐ 2. Rather not serious

☐ 3. Moderately serious

☐ 4. Rather serious

☐ 5. Very serious

9. Are you afraid of contracting Lyme disease?

☐ 1. Definitely not

☐ 2. Rather not

☐ 3. I don’t know

☐ 4. Rather yes

☐ 5. Definitely yes

10. What do you think is your risk of contracting Lyme disease?

☐ 1. Very low

☐ 2. Rather low

☐ 3. Average

☐ 4. Rather high

☐ 5. Very high

11. If you were vaccinated against Lyme disease, would you be less afraid of this disease?

☐ 1. Definitely not

☐ 2. Rather not

☐ 3. I don’t know

☐ 4. Rather yes

☐ 5. Definitely yes

12. Do you believe that protective measures such as using tick repellents, wearing appropriate clothing, and carefully checking your body after outdoor activities are enough to prevent Lyme disease?

☐ 1. Definitely not

☐ 2. Rather not

☐ 3. I don’t know

☐ 4. Rather yes

☐ 5. Definitely yes

13. Do you think that the availability of effective antibiotic treatments for Lyme disease is a sufficient reason to forgo vaccination?

☐ 1. Definitely not

☐ 2. Rather not

☐ 3. I don’t know

☐ 4. Rather yes

☐ 5. Definitely yes

14. How necessary do you think a vaccine for Lyme disease is?

☐ 1. Completely unnecessary

☐ 2. Rather unnecessary

☐ 3. Moderately necessary

☐ 4. Rather necessary

☐ 5. Very necessary

15. Which aspects of a future Lyme disease vaccine are important to you?

I. Confirmed effectiveness of at least 90%.

☐ 1. Not important

☐ 2. Rather unimportant

☐ 3. Moderately important

☐ 4. Rather important

☐ 5. Very important

II. Confirmed safety based on research.

☐ 1. Not important

☐ 2. Rather unimportant

☐ 3. Moderately important

☐ 4. Rather important

☐ 5. Very important

III. Long-term experience with the vaccine in the general population.

☐ 1. Not important

☐ 2. Rather unimportant

☐ 3. Moderately important

☐ 4. Rather important

☐ 5. Very important

IV. Long-lasting effects/no need for booster doses.

☐ 1. Not important

☐ 2. Rather unimportant

☐ 3. Moderately important

☐ 4. Rather important

☐ 5. Very important

V. Availability at family doctor's offices.

☐ 1. Not important

☐ 2. Rather unimportant

☐ 3. Moderately important

☐ 4. Rather important

☐ 5. Very important

VI. Possibility to get vaccinated at a pharmacy.

☐ 1. Not important

☐ 2. Rather unimportant

☐ 3. Moderately important

☐ 4. Rather important

☐ 5. Very important

VII. Funding by NFZ (free vaccination).

☐ 1. Not important

☐ 2. Rather unimportant

☐ 3. Moderately important

☐ 4. Rather important

☐ 5. Very important

16. Would you be willing to get vaccinated if the Lyme disease vaccine met your requirements?

☐ 1. Definitely not

☐ 2. Rather not

☐ 3. I don’t know

☐ 4. Rather yes

☐ 5. Definitely yes

17. Would you vaccinate your child if a Lyme disease vaccine was available for children?

☐ 1. Definitely not

☐ 2. Rather not

☐ 3. I don’t know

☐ 4. Rather yes

☐ 5. Definitely yes

18. If a Lyme disease vaccine was paid, what is the maximum amount you would be willing to pay for a full vaccination cycle (all doses)?

☐ 1. Less than 100 zł

☐ 2. 100–299 zł

☐ 3. 300–499 zł

☐ 4. 500–999 zł

☐ 5. 1000 zł or more

19. Do you believe that the risks associated with vaccination are greater than the risks of contracting Lyme disease?

☐ 1. Definitely not

☐ 2. Rather not

☐ 3. I don’t know

☐ 4. Rather yes

☐ 5. Definitely yes

20. Is fear of needles a reason why you are hesitant about vaccinations?

☐ 1. Definitely not

☐ 2. Rather not

☐ 3. I don’t know

☐ 4. Rather yes

☐ 5. Definitely yes

21. Are you afraid of the side effects of vaccines?

☐ 1. Definitely not

☐ 2. Rather not

☐ 3. I don’t know

☐ 4. Rather yes

☐ 5. Definitely yes

22. Do you trust experts (doctors, pharmacists, scientists) who recommend vaccinations?

☐ 1. Definitely not

☐ 2. Rather not

☐ 3. I don’t know

☐ 4. Rather yes

☐ 5. Definitely yes

23. What is your general attitude towards vaccinations?

☐ 1. Strongly negative

☐ 2. Rather negative

☐ 3. Neutral

☐ 4. Rather positive

☐ 5. Strongly positive

24. Gender:

☐ 1. Male

☐ 2. Female

25. Age:

☐ 1. 18–24

☐ 2. 25–34

☐ 3. 35–44

☐ 4. 45 and above

26. How many children do you have?

☐ 1

☐ 2

☐ 3

☐ 4 or more

27. Place of residence:

☐ 1. Village

☐ 2. City with less than 50,000 residents

☐ 3. City with 50,000–100,000 residents

☐ 4. City with 100,000–500,000 residents

☐ 5. City with more than 500,000 residents

28. Education level:

☐ 1. Primary

☐ 2. Vocational

☐ 3. Secondary

☐ 4. Higher

29. How would you rate your financial situation?

☐ 1. We are wealthy and can afford any expense.

☐ 2. We are fairly well-off but need to save for larger expenses.

☐ 3. Average, we are neither poor nor wealthy.

☐ 4. We are fairly poor but can manage.

☐ 5. We are poor, and every expense is a problem.
